# Flavonoids from *Epimedium pubescens*: extraction and mechanism, antioxidant capacity and effects on CAT and GSH-Px of *Drosophila melanogaster*

**DOI:** 10.7717/peerj.8361

**Published:** 2020-01-20

**Authors:** Xiao-Hua Yang, Lu Li, Yao-Bi Xue, Xue-Xue Zhou, Jie-Hua Tang

**Affiliations:** 1 Health Science Center, Xi’an Jiaotong University, Xi’an, the People’s Republic of China; 2 International Joint Research Center of Shaanxi Province for Food and Health Sciences, National Engineering Laboratory for Resources Development of Endangered Crude Drugs in Northwest China, College of Food Engineering and Nutritional Science, Shaanxi Normal University, Xi’an, the People’s Republic of China

**Keywords:** Flavonoids, *Epimedium pubescens*, Ultrasonic extraction, Antioxidant property, *Drosophila melanogaster*

## Abstract

**Background:**

*Epimedium* is a famous medicinal plant in China, Southeast Asian and some other regions. Flavonoids are regarded as its supremely important active constituents used in phytomedicines and/or functional foods. It is of theoretical and applied significance to optimize the procedure for extraction of flavonoids with high bioactivity from *Epimedium*, to unveil extraction mechanism, to identify chemical composition of flavonoids, to analyze free radical-scavenging ability of flavonoids, and to investigate their effects on the model organism *Drosophila melanogaster*.

**Methods:**

Box-Behnken design was applied to optimization of extraction procedure. Laser diffraction particle size analysis was used to clarify extraction mechanism. Chemical composition of flavonoids was analyzed using high-performance liquid chromatography. Antiradical capacities of flavonoids were determined by chemical-based assay. Then, effects of flavonoids on catalase (CAT) and glutathione peroxidase (GSH-Px) in *D. melanogaster* were investigated for the first time.

**Results:**

The optimal condition for ultrasonic extraction of antioxidant flavonoids from *Epimedium pubescens* was achieved and extraction mechanism was discussed. *Epimedium* flavonoids contained icariin, epimedin A, epimedin B and epimedin C. *Epimedium* flavonoids exhibited the ability to scavenge ABTS^+^ and DPPH^⋅^ radicals with EC_50_ values of 55.8 and 52.1 µg/ml, respectively. Moreover, *Epimedium* flavonoids were able to increase activities of CAT and GSH-Px in *D. melanogaster*. For females, oral administration of flavonoids improved CAT and GSH-Px activities by 13.58% and 5.18%, respectively. For males, oral administration of flavonoids increased CAT and GSH-Px activities by 13.90% and 5.65%, respectively.

**Conclusion:**

Flavonoids ultrasonically extracted from *E. pubescens* considerably affected antioxidant defense system in *D. melanogaster*. Flavonoids of *E. pubescens* showed great potential for becoming a natural antioxidant because of their antiradical ability and effects on CAT and GSH-Px of the model organism.

## Introduction

*Epimedium* L., a genus of Berberidaceae family, is frequently used as medicinal plant in China, Japan, Southeast Asian and Mediterranean region (Zhang et al., 2014; Yang et al., 2018). The genus comprises approximately 58 species (Zhang et al., 2016), and five of them (*Epimedium pubescens, E. sagittatum, E. brevicornum, E. koreanum* and *E. wushanense*) are included in Pharmacopoeia of the People’s Republic of China (The State Pharmacopoeia Commission of the People’s Republic of China, 2015). *E*. *pubescens* has been applied to herbal preparations and functional foods in China for more than 2000 years (Zhang & Yang, 2012; Yang et al., 2018). Usually, *Epimedium* is used for prevention and treatment of diseases of reproductive system, nervous system, endocrine system and immune system (Wu, Lien & Lien, 2003; Zhang et al., 2008; Zhang & Yang, 2012). *Epimedium* contains a vast array of nutrients and phytochemicals, such as protein, essential elements, polysaccharides, flavonoids, alkaloids, lignans and terpenoids (Wu, Lien & Lien, 2003; Zhang & Yang, 2010). Among them, flavonoids are considered the most important active constituents, which possess efficacy on hematopoietic system, cardiovascular and cerebrovascular systems, internal secretion and immune systems, reproductive system, and nervous system (Jin et al., 2019; Shen & Wang, 2018; Li et al., 2015; Maraldi et al., 2019). Meanwhile, *Epimedium* flavonoids exhibit anti-osteoporosis, anti-tumor (e.g., blood cancer), anti-aging and anti-inflammatory activities (Zhang & Yang, 2010; Yang et al., 2018; Yang, Xi & Li, 2019; Wang et al., 2018). Besides, the major flavonoids of *Epimedium* (e.g., epimedin A, epimedin B and icariin) have no acute toxicity and long-term adverse health effects (Li et al., 2008; Hwang et al., 2017; Zhong et al., 2019; Ling et al., 2018). As a result, there is an increasing interest in *Epimedium* flavonoids for developing phytomedicines and/or functional foods (Zhang et al., 2014; Yang et al., 2018).

*Epimedium* contains about 70 kinds of flavonoids, many of which possess pharmacological activity and health benefits (Wang, Tsai & Lin, 2007; Zhang & Yang, 2010; Li et al., 2012). In many cases, a single flavonoid does not display potent bioactivity individually, and the combination of some flavonoids may produce synergistic effects (Wang, Tsai & Lin, 2007; Zhang & Yang, 2010; Tu, 2011). Therefore, it is of interest to optimize the procedure for extraction of the combination of flavonoids with high bioactivity from *Epimedium*. It is well known that release of target compounds (e.g., flavonoids) from vegetal matrix is generally one of the most important steps of utilization of resources of edible medicinal plants (Vinatoru, 2001; Wang & Weller, 2006). In the past two decades, a wide variety of techniques, such as heating extraction, percolation extraction, Soxhlet extraction, microwave assisted extraction and supercritical fluid extraction, were used to isolate flavonoids from *Epimedium* (Zhang, Yang & Wang, 2011). Alternatively, ultrasonic extraction is regarded as a powerful tool for extracting flavonoids from plant samples (Kazemi, Khodaiyan & Hosseini, 2019). Compared to conventional techniques such as Soxhlet extraction, ultrasonic extraction frequently shows distinctive advantages in higher extraction yield, shorter extraction time and lower extraction temperature (Luque-García & Luque de Castro, 2003; Wang & Weller, 2006). Meanwhile, compared with microwave assisted extraction and supercritical fluid extraction, ultrasonic extraction usually requires simpler equipment and less severe operational condition (Luque-García & Luque de Castro, 2003; Hu et al., 2007). Some earlier studies have dealt with ultrasonic extraction of total flavonoids and specific compounds (e.g., icariin) from *Epimedium* (Zhang et al., 2008; Zhang, Yang & Wang, 2011). However, there is almost no research into the mechanism underlying ultrasonic extraction of flavonoids from *E. pubescens* by laser diffraction particle size analysis.

Growing evidence indicates that the balance between reactive oxygen species (ROS) production and antioxidant defense plays important roles in human health (Finkel & Holbrook, 2000; Shebis et al., 2013). In general, antioxidant defense system includes enzymatic scavengers and non-enzymatic molecules (e.g., ascorbic acid) (Shebis et al., 2013). Two enzymatic scavengers, catalase (CAT, EC 1.11.1.6) and glutathione peroxidase (GSH-Px, EC 1.11.1.9), can protect cells from oxidative injury caused by peroxides (Shebis et al., 2013). When the balance is drastically disturbed, some health problems such as cancer, aging, Alzheimer’s and Parkinson’s diseases, may occur (Finkel & Holbrook, 2000; Yang et al., 2017). During alcohol intoxication, dependence and withdrawal, activities of antioxidant enzymes such as CAT generally decreased and ROS production accordingly increased, which might lead to alcohol-induced brain damage (Haddadi et al., 2014; Deepashree et al., 2019). Consequently, it is important to search for new natural antioxidants and to assess their protective effect against oxidative damage (Shebis et al., 2013). Traditionally, *Epimedium* was used to prevent and treat several diseases such as sexual dysfunction, osteoporosis, amnesia and rheumatism, some of which were intimately linked with oxidative stress (Yang et al., 2017). Antioxidant capacity of natural compounds is routinely evaluated by chemical-based assay, cellular-based assay, animal assay and human assay (López-Alarcón & Denicola, 2013). Chemical-based assay presents great merits in low cost, high throughput and short duration (Yang et al., 2017). However, the results obtained by chemical-based assay cannot accurately express *in vivo* antioxidant capacity due to the complexity of organism (López-Alarcón & Denicola, 2013).

In recent years, *Drosophila melanogaster* (fruit fly) is often employed as a model organism for researches into oxidative stress because of its obvious advantages in small body size, short lifespan and easy maintenance (Peng et al., 2011). Additionally, the overwhelming majority of human disease genes have corresponding DNA sequences in *D. melanogaster* (Kaun, Devineni & Heberlein, 2012), which provides an opportunity to study certain diseases related to oxidative stress. To date, *D. melanogaster* has been successfully used to assess *in vivo* antioxidant property, lifespan-prolonging activity and toxicity of several natural products such as apple polyphenols, chlorogenic acid, quercetin and kaempferol (Peng et al., 2011; Sotibrán, Ordaz-Téllez & Rodríguez-Arnaiz, 2011). Nevertheless, there is no report on the application of *D. melanogaster* to *in vivo* antioxidant capacities of flavonoids from *E. pubescens*.

The aims of the present study are to optimize ultrasonic extraction of the combination of flavonoids from *E. pubescens* using response surface methodology (RSM), to clarify extraction mechanism by laser diffraction particle size analysis, and to characterize chemical composition of *Epimedium* flavonoids. Then free radical-scavenging ability of *Epimedium* flavonoids is analyzed by DPPH (1,1-diphenyl-2-picrylhydrazyl) and ABTS (2,2′-azino-bis (3-ethyl-benzothiazoline-6-sulfonic acid) diammonium salt) methods, and their effects on CAT and GSH-Px in *D. melanogaster* are investigated for the first time.

## Materials and Methods

### Electrical apparatus

A Breeze 1,525 high-performance liquid chromatography (HPLC) system equipped with a Breeze 2,487 ultraviolet–visible detector (Waters Corporation, USA) and a ZORBAX SB-C8 reverse-phase analytical column (Agilent Technologies, USA) was applied to separation and quantification of icariin, epimedin A, epimedin B and epimedin C. A JPCQ0328 ultrasonic cleaning bath (Wuhan Jiapeng Electronics Co., Ltd., China) working at 120 W power was employed as the device for ultrasonic extraction. A TU-1810 ultraviolet–visible spectrophotometer (Beijing Purkinje General Instrument Co., Ltd., China) was used for determination of total flavonoids, radical-scavenging capacities and antioxidant enzyme activities. An HWS-150B electrothermal constant temperature incubator (Tianjin Taisite Instrument Co., Ltd., China) was used to rear fruit flies. A Mastersizer 2000 laser diffraction particle size analyzer (Malvern Instrument Ltd., UK) was applied to clarification of extraction mechanism. Other related apparatus and accessories included an RE-52 rotary evaporator (Anting Scientific Instrument Co., Ltd., China), a LGJ-18C vacuum freeze drier (Sihuan Scientific Instrument Co., Ltd., China), an XTL-201 stereo microscope (Guangzhou Liss Optical Instrument Co., Ltd., China), an L420 low-speed centrifuge (Hunan Xiangyi Laboratory Instrument Co., Ltd., China), an FW400A high-speed grinder (Beijing Kewei Yongxing Instrument Co., Ltd., China), and a BS224S analytical balance (Sartorius AG, Germany).

### Plant materials and chemicals

The leaves of *E. pubescens* were collected from Hubei Province of China, and taxonomically identified by one of the authors (X.-H. Yang). The leaves were air-dried at room temperature and then milled into powders. The powders were oven-dried to constant weight at 55–60 °C, and then stored in a dark vessel at room temperature until use.

Standards of epimedin A, epimedin B and epimedin C (Purity ≥ 99%) were purchased from ChromaDex, Inc. (USA). Standards of icariin and vitamin C (Vc) (Purity ≥ 98%) were bought from Shanghai Yuanye Bio-Technology Co., Ltd. (China). DPPH, ABTS and bovine serum albumin were purchased from Sigma Chemical Co. (USA). Acetonitrile (HPLC grade) was bought from Fisher Chemicals (USA). Enzyme assay kits (including catalase assay kit and glutathione peroxidase assay kit) were purchased from Nanjing Jiancheng Bioengineering Institute (China). Other chemicals such as ethanol and glacial acetic acid (analytical grade) were purchased from Tianjin Tianli Chemicals Co., Ltd. (China).

### Extraction procedure

#### Ultrasonic extraction

Leaf powders of 0.5 g were mixed with special volume (5, 10, 15, 30, 35 ml) of aqueous ethanol solution at certain concentration (0, 20%, 40%, 60%, 80%, 100%) in a flask, and marinated at room temperature for 30 min. Afterwards, the flask was placed in the ultrasonic cleaning bath, and leaf powders were extracted at certain temperature (15, 25, 30, 35, 45, 55 °C) for special time (10, 20, 30, 40, 60 min). In the process of extraction, the position of the flask was changed randomly at regular intervals to ensure homogenous exposure of the mixture of leaf powders and ethanol solution to ultrasound irradiation. After ultrasonication, the mixture was centrifuged at 4000 rpm for 10 min, and the supernatants were filtered through 0.45 µm microporous membranes. The filtrates were concentrated by the rotary evaporator under vacuum and then lyophilized at −48 °C until the solvent was completely removed. Extraction yield of flavonoids from *E. pubescens* is calculated using the following equation:

(1) \begin{eqnarray*}\mathrm{Y }(\text{%})={\mathrm{W}}_{\mathrm{f}}\div {\mathrm{W}}_{\mathrm{l}}\times 100\text{%}\end{eqnarray*}

where Y is extraction yield of flavonoids, W_f_ is the weight of flavonoids (mg), and W_l_ is the weight of leaf powders (mg).

### Heating extraction

Leaf powders of 0.5 g were mixed with 63% (v/v) ethanol solution of 35 ml, steeped at room temperature for 30 min, and heated at 38 °C for 39 min. Then the mixture of leaf powders and ethanol solution was spun at 4,000 rpm for 10 min. The supernatants were filtrated through microporous membranes, evaporated under vacuum and finally freeze-dried. Extraction yield of *Epimedium* flavonoids is calculated as described above.

### Identification and quantification of flavonoids

HPLC method was used to separate and quantify *Epimedium* flavonoids (Zhang et al., 2008). The chromatographic peaks of flavonoids were confirmed based upon spectral characteristics, retention time, and co-injection of the samples with standards. And spectrophotometric method was applied to determination of total flavonoids, as icariin equivalents (The State Pharmacopoeia Commission of the People’s Republic of China, 2015).

### Particle size analysis

Particle size analysis was performed as described by Kwak et al. (2009) with slight modification. Briefly, *Epimedium* samples processed by various extraction methods (i.e., ultrasonic extraction and heating extraction) were oven-dried at 45–50 °C. The dried samples were dispersed to deionized water and particle size distribution was measured. The refractive indices of the samples and water were 1.33 and 1.59, respectively.

### Measurement of antiradical activity

#### ABTS^⋅+^ radical-scavenging activity

Modified method of Yang et al. (2017) were used to determine ABTS^⋅+^ radical-scavenging capacity. ABTS^⋅+^ radical solution was prepared using the reaction between ABTS and potassium persulfate. Flavonoid solutions at different concentrations (10, 60, 110, 160, 210, 260, 310 µg/ml) of 1.0 ml were respectively mixed with ABTS^⋅+^ radical solution of 4.0 ml, and incubated in the dark at 30 °C for 6 min. Immediately, the mixtures were tested for the absorbance at 734 nm. In negative and positive controls, ultrapure water and Vc solution were used instead of flavonoid solution, respectively. ABTS^⋅+^ radical-scavenging capacity is calculated using the following equation:

(2) \begin{eqnarray*}\mathrm{RA}(\text{%})=({\mathrm{A}}_{\mathrm{n}}-{\mathrm{A}}_{\mathrm{f}})\div {\mathrm{A}}_{\mathrm{n}}\times 100\text{%}\end{eqnarray*}

where RA (%) is radical-scavenging activity, A_f_ is the absorbance of the mixture of ABTS^⋅+^ radical solution and flavonoid solution, and A_n_ is the absorbance of negative control. The value of EC_50_ (effective concentration that reduced chemiluminescence by 50%) is calculated according to Shahidi & Zhong (2015). All experiments were carried out in triplicate, and the results of ABTS assay were plotted with means of three replicates.

#### DPPH^⋅^ radical-scavenging activity

DPPH assay was modified from Yang et al. (2017). Flavonoid solutions of 1.0 ml at various concentrations (10, 60, 110, 160, 210, 260, 310 µg/ml) were respectively mixed with DPPH solution of 2.0 ml, and incubated in the dark at 37 °C for 30 min. The absorbance of the mixture was recorded at 517 nm. Ultrapure water and Vc solution were used in negative and positive controls, respectively. DPPH^⋅^ radical-scavenging capacity is calculated using the following equation:

(3) \begin{eqnarray*}\mathrm{RD}(\text{%})=({\mathrm{A}}_{\mathrm{n}}-{\mathrm{A}}_{\mathrm{f}})\div {\mathrm{A}}_{\mathrm{n}}\times 100\text{%}\end{eqnarray*}

where RD (%) is radical-scavenging activity, A_f_ is the absorbance of the mixture of DPPH solution and flavonoid solution, and A_n_ is the absorbance of negative control. EC_50_ value is calculated as described above. The results of DPPH assay were also plotted with means of replicates.

### Animal experiments

#### Drosophila melanogaster strain and culture condition

Animal experiments were conducted as described with slight modification (Peng et al., 2011; Deepashree et al., 2019). The study was reviewed and approved by the Ethics Committee of Health Science Center, Xi’an Jiaotong University (Protocol no.: 2017-375). And wild-type fruit flies used for all *in vivo* experiments were provided by Health Science Center, Xi’an Jiaotong University. Newly eclosed fruit flies (1- to 2-day-old) were randomly divided into four groups (*n* = 20): male control group; female control group; male experiment group; and female experiment group. Fruit flies in the control groups were fed on the cornmeal medium (10.0% of cornmeal, 1.5% of agar powders, 13.5% of sugar, 0.5% of propanoic acid, 1.0% of dried yeast powders and 73.5% of deionized water). And fruit flies in the experiment groups were raised on the flavonoid medium, which was actually the cornmeal medium supplemented with *Epimedium* flavonoids (final concentration of 0.5 mg/ml). All fruit flies were reared for 20 days under a L12:D12 photoperiod (12 h light/dark cycle), at relative humidity of 60% and temperature of 25 °C. Afterwards, they were frozen to death at −20 °C for further test.

#### Preparation of fruit fly homogenates

According to the instructions of commercial enzyme assay kits, fruit flies were homogenized on ice in the pre-chilled kit buffer at a ratio of 1.0 g fruit flies per 33 ml, and immediately centrifuged at 3,000 rpm for 15 min at 4 °C. The supernatants were collected as fruit fly homogenates. Contents of protein in the homogenates were determined by the Bradford method (Bradford, 1976).

#### Determination of antioxidant enzyme activities

##### CAT assay.

Fruit fly homogenates were analyzed for CAT activity using a commercial catalase assay kit according to the manufacturer’s protocols. The color reaction between molybdate and H_2_O_2_ in the presence of CAT was spectrophotometrically monitored at 405 nm. CAT activity is calculated using the following equation:

(4) \begin{eqnarray*}\mathrm{AC}(\mathrm{U/ mg} \mathrm{protein})=({\mathrm{A}}_{\mathrm{c}}-{\mathrm{A}}_{\mathrm{s}})\times 271\div (60\times \mathrm{V })\div {\mathrm{C}}_{\mathrm{p}}\end{eqnarray*}

where AC is enzyme activity, A_c_ is the absorbance of control (in the absence of fruit fly homogenates), A_s_ is the absorbance of samples (in the presence of fruit fly homogenates), V is the volume of samples used to detect (ml), and C_p_ is protein content of samples (mg/ml).

##### GSH-Px assay.

Analysis of GSH-Px activity was carried out according to the manufacturer’s instructions of glutathione peroxidase assay kit. Conversion of 5,5′-dithiobis (2-nitro-benzoic acid) to 2-nitro-5-thiobenzoic acid in the presence of glutathione (GSH) and GSH-Px was monitored at 412 nm. GSH-Px activity is calculated using the following equation:

(5) \begin{eqnarray*}\mathrm{AG}(\mathrm{U/ mg} \mathrm{protein})=({\mathrm{A}}_{\mathrm{c}}-{\mathrm{A}}_{\mathrm{sa}})\div ({\mathrm{A}}_{\mathrm{st}}-{\mathrm{A}}_{\mathrm{b}})\times {\mathrm{C}}_{\mathrm{st}}\times \mathrm{D}\div \mathrm{T}\div ({\mathrm{C}}_{\mathrm{p}}\times \mathrm{V })\end{eqnarray*}

where AG is enzyme activity, A_c_ is the absorbance of control (fruit fly homogenates are added after incubation in the presence of GSH), A_sa_ is the absorbance of samples (fruit fly homogenates are added before incubation in the presence of GSH), A_st_ is the absorbance of standard GSH (in the absence of fruit fly homogenates), A_b_ is the absorbance of blank (in the absence of GSH and fruit fly homogenates), C_st_ is content of standard GSH (µmol/l), D is dilution factor, T is reaction time (min), C_p_ is protein content of samples (mg/ml), and V is the volume of samples used to detect (ml).

### Computer software and statistical analysis

The optimum condition for extraction of flavonoids from *E. pubescens* was achieved by RSM, which was implemented using Design-Expert trial version 7.0 software (Stat-Ease Inc., USA). A Box-Behnken design (BBD) was used to investigate effects of four independent variables (ultrasonication time, ethanol concentration, liquid to solid ratio and extraction temperature) at three levels on extraction yield. Code and levels of variables used in the BBD were listed in Table 1. The complete design consisted of 29 experimental runs (Table 2). All experiments were performed in random order. The adequacy of model was checked by “R^2^” and “adjusted R^2^ (adj-R^2^)”. The reliability of equation was evaluated by “lack of fit”. The relationship between two variables was illustrated in three dimensional (3D) response surface plots and contour plots based on regression equation. Two variables were depicted in one 3D plot whilst the other variables were kept at zero level (0 level).

**Table 1:** Code and levels of extraction variables used in Box-Behnken design.

Extraction variable	Coded symbol	Coded level		
		−1	0	1
Ultrasonication time (min)	X_1_	20	30	40
Liquid to solid ratio (ml/g)	X_2_	50	60	70
Ethanol concentration (%)	X_3_	40	60	80
Extraction temperature (°C)	X_4_	20	30	40

**Table 2:** Box-Behnken design and response values observed.

Run	Extraction variable				Response value (Extraction yield, %)
	X_1_	X_2_	X_3_	X_4_	
1	−1 (20)	0 (60)	0 (60)	1 (40)	9.296
2	0 (30)	0 (60)	−1 (40)	1 (40)	8.199
3	0 (30)	0 (60)	0 (60)	0 (30)	8.921
4	−1 (20)	1 (70)	0 (60)	0 (30)	8.966
5	−1 (20)	0 (60)	1 (80)	0 (30)	8.268
6	0 (30)	0 (60)	1 (80)	−1 (20)	7.657
7	1 (40)	1 (70)	0 (60)	0 (30)	9.257
8	0 (30)	0 (60)	0 (60)	0 (30)	8.685
9	0 (30)	1 (70)	1 (80)	0 (30)	8.641
10	1 (40)	0 (60)	1 (80)	0 (30)	8.546
11	0 (30)	0 (60)	1 (80)	1 (40)	8.699
12	0 (30)	−1 (50)	0 (60)	1 (40)	8.962
13	0 (30)	0 (60)	0 (60)	0 (30)	8.990
14	−1 (20)	0 (60)	0 (60)	−1 (20)	8.615
15	−1 (20)	0 (60)	−1 (40)	0 (30)	7.963
16	0 (30)	1 (70)	0 (60)	1 (40)	9.095
17	1 (40)	0 (60)	0 (60)	1 (40)	9.074
18	1 (40)	0 (60)	0 (60)	−1 (20)	8.699
19	1 (40)	0 (60)	−1 (40)	0 (30)	7.824
20	0 (30)	−1 (50)	1 (80)	0 (30)	8.267
21	1 (40)	−1 (50)	0 (60)	0 (30)	8.661
22	0 (30)	1 (70)	−1 (40)	0 (30)	7.831
23	−1 (20)	−1 (50)	0 (60)	0 (30)	8.846
24	0 (30)	0 (60)	0 (60)	0 (30)	8.782
25	0 (30)	0 (60)	0 (60)	0 (30)	8.532
26	0 (30)	−1 (50)	0 (60)	−1 (20)	8.395
27	0 (30)	0 (60)	−1 (40)	−1 (20)	7.629
28	0 (30)	1 (70)	0 (60)	−1 (20)	8.463
29	0 (30)	−1 (50)	−1 (40)	0 (30)	7.781

In this study, every experiment was repeated at least twice (2–3 times). All data were expressed as mean ± SD (standard deviation) of multiple replicates unless otherwise stated. One-way ANOVA (analysis of variance) was applied to determination of differences among means using SPSS statistical software (SPSS Inc., USA). Differences were considered significant and very significant when 0.01 ≤ *p* < 0.05 and *p* < 0.01, respectively.

## Results and Discussion

### Optimization of extraction condition and clarification of extraction mechanism

#### Influences of extraction parameters on extraction yield

**Figure 1:**
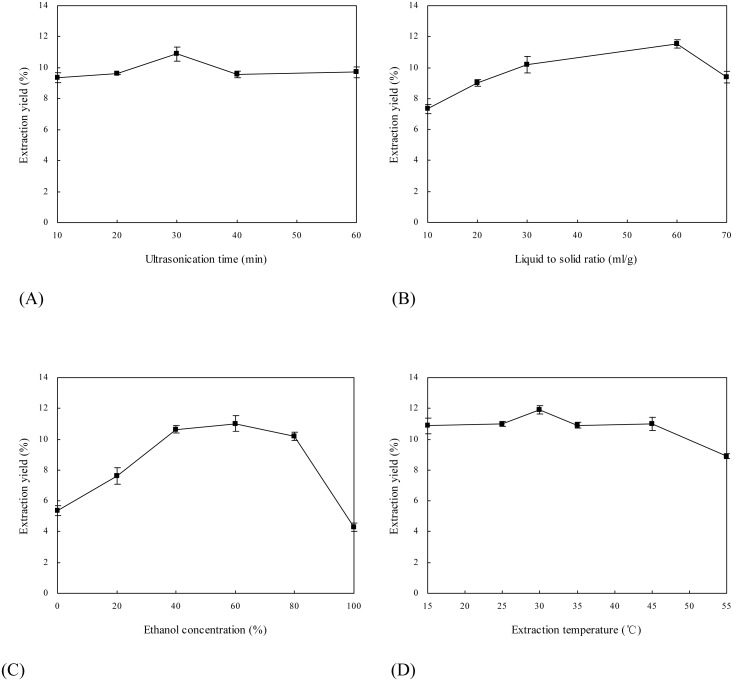
Effects of extraction parameters on extraction yield of flavonoids from *Epimedium*. (A) Ultrasonication time; (B) Liquid to solid ratio; (C) Ethanol concentration; (D) Extraction temperature.

It is known that ultrasonic extraction of bioactive components from plants may be affected by a great number of factors, such as ultrasound frequency, ultrasonication time, liquid to solid ratio, extractant and extraction temperature (Zhang et al., 2008; Kazemi, Khodaiyan & Hosseini, 2019). Because low frequency of ultrasound was helpful to the improvement of extraction yield of target compounds and the degradation of toxic alkaloids in many cases (Vinatoru, 2001), 30 kHz was chosen as the working frequency. Effects of four extraction parameters (i.e., ultrasonication time, liquid to solid ratio, ethanol concentration and extraction temperature) on extraction yield of flavonoids from *E. pubescens* were systematically investigated (Fig. 1).

Figure 1A illustrates influence of ultrasonication time on extraction yield of flavonoids from *E. pubescens*. Extraction yield increased with extending ultrasonication time from 10 to 30 min, and decreased thereafter. When ultrasound travels in the liquid, it may create bubbles or cavities (Luque-García & Luque de Castro, 2003). The process by which bubbles or cavities form, grow and collapse is called acoustic cavitation. Acoustic cavitation possesses physical and chemical effects (Wang & Weller, 2006). The physical effects include the production of high-speed jets of liquid, which may result in the disruptions of *Epimedium* samples and the efficient exudation of flavonoids out of the matrix. However, with the prolongation of ultrasonication time, more and more free radicals are generated by sonolysis of solvent (Qiao et al., 2014), which may interact with *Epimedium* flavonoids and induce their degradation.

Effect of liquid to solid ratio on extraction yield of *Epimedium* flavonoids is shown in Fig. 1B. With the increase of liquid to solid ratio from 10 to 60 ml/g, extraction yield improved gradually. The highest extraction yield was obtained when liquid to solid ratio reached 60 ml/g. This is probably due to the fact that a larger volume of ethanol solution generally dissolves a larger quantity of flavonoids. However, extraction yield declined significantly (*p* < 0.05) while liquid to solid ratio was more than 60 ml/g. Likewise, Xia et al. (2011) observed that extraction yield of phillyrin from *Forsythia suspensa* at first increased and then reduced with improving liquid to solid ratio from 5 to 30 ml/g. One of the possible reasons for the lower extraction yield of flavonoids with the higher liquid to solid ratio is that more flavonoids are degraded by exposure to ultrasound irradiation when flavonoids are dispersed in excessive amount of ethanol solution (Luque-García & Luque de Castro, 2003).

Figure 1C reveals that ethanol concentration greatly affected extraction yield of *Epimedium* flavonoids. Extraction yield of flavonoids notably rose as ethanol concentration increased from 0% to 40% (v/v) (*p* < 0.01), and then levitated in the range of 40%–60% (*p* > 0.05). Extraction yield achieved a maximum at ethanol concentration of 60%, and hereafter decreased with the increase of ethanol concentration from 60% to 100%. The possible reasons include (1) *Epimedium* samples are prone to be hydrated with 60% ethanol solution; (2) 60% ethanol solution has relatively great affinity for flavonoids; and (3) flavonoids have relatively high solubility in 60% ethanol solution (Zhang et al., 2013).

Influence of extraction temperature on extraction yield of *Epimedium* flavonoids is illustrated in Fig. 1D. The highest extraction yield was achieved at 30 °C. And extraction yield declined at lower or higher temperatures. In the range of 15–30 °C, relatively high temperature may increase the number of bubbles or cavities formed by ultrasound and reduce the viscosity of ethanol solution, which facilitate mass transfer between flavonoids and ethanol solution and subsequently increase extraction yield. In the range of 30–55 °C, extremely high temperature may reduce the number of bubbles or cavities and attenuate the impact of acoustic cavitation on *Epimedium* samples (Palma & Barroso, 2002).

#### BBD

To understand the interaction between extraction variables in a consecutive range and to further optimize the condition for ultrasonic extraction of flavonoids from *E. pubescens*, response surface methodology was carried out depending on the results of the above single factor experiments (Li et al., 2019). The four factors in BBD included ultrasonication time (X_1_, 20–40 min), liquid to solid ratio (X_2_, 50–70 ml/g), ethanol concentration (X_3_, 40%–80%) and extraction temperature (X_4_, 20–40 °C) (Table 1). The results of 29 experimental runs in BBD were presented in Table 2 and the statistical data was shown in Table A.1. R^2^ is 0.9494, suggesting that BBD model is capable of elucidating the interaction of selected variables; and adj-R^2^ is higher than 0.8 (adj- *R*^2^ = 0.8988), indicating that the model do not include non-significant terms (Mahfoudhi et al., 2014). It means that there is a high degree of correlation between the observed and predicted values, and the fitness and reliability of the model are satisfactory. On the basis of regression analysis, BBD model can be expressed by the following equation:

(6) \begin{eqnarray*}Y=6.8058-0.1546{\mathrm{X}}_{1}-0.0779{\mathrm{X}}_{2}+0.1680{\mathrm{X}}_{3}+0.0268{\mathrm{X}}_{4}+0.0012{\mathrm{X}}_{1}{\mathrm{X}}_{2}+0.0005{\mathrm{X}}_{1}{\mathrm{X}}_{3}\nonumber\\\displaystyle -0.0008{\mathrm{X}}_{1}{\mathrm{X}}_{4}+0.0004{\mathrm{X}}_{2}{\mathrm{X}}_{3}+0.0002{\mathrm{X}}_{2}{\mathrm{X}}_{4}+0.0006{\mathrm{X}}_{3}{\mathrm{X}}_{4}+0.0013{\mathrm{X}}_{1}^{2}+0.0002{\mathrm{X}}_{2}^{2}-\nonumber\\\displaystyle 0.0018{\mathrm{X}}_{3}^{2}-0.0003{\mathrm{X}}_{4}^{2}\end{eqnarray*}

where Y is response values and X_i_ (*i* = 1, 2, 3 and 4) is the coded variables. According to Eq. (6), response values were related to the coded variables by the second-order polynomial. According to F and *p* values, extraction variables possessing significant effects on extraction yield were the quadratic term of ethanol concentration (${X}_{3}^{2}$), followed by the linear terms of extraction temperature (X_4_), ethanol concentration (X_3_) and liquid to solid ratio (X_2_) (Table A.1). On the contrary, the linear term of ultrasonication time (X_1_), the quadratic term of ultrasonication time (${X}_{1}^{2}$), the quadratic term of liquid to solid ratio (${X}_{2}^{2}$) and the quadratic term of extraction temperature (${X}_{4}^{2}$) and the interaction terms of two variables (*X*_1_*X*_2_, *X*_1_*X*_3_, *X*_1_*X*_4_, *X*_2_*X*_3_, *X*_2_*X*_4_ and *X*_3_*X*_4_) were found to be not significant (*p* > 0.05) (Table A.1).

**Figure 2:**
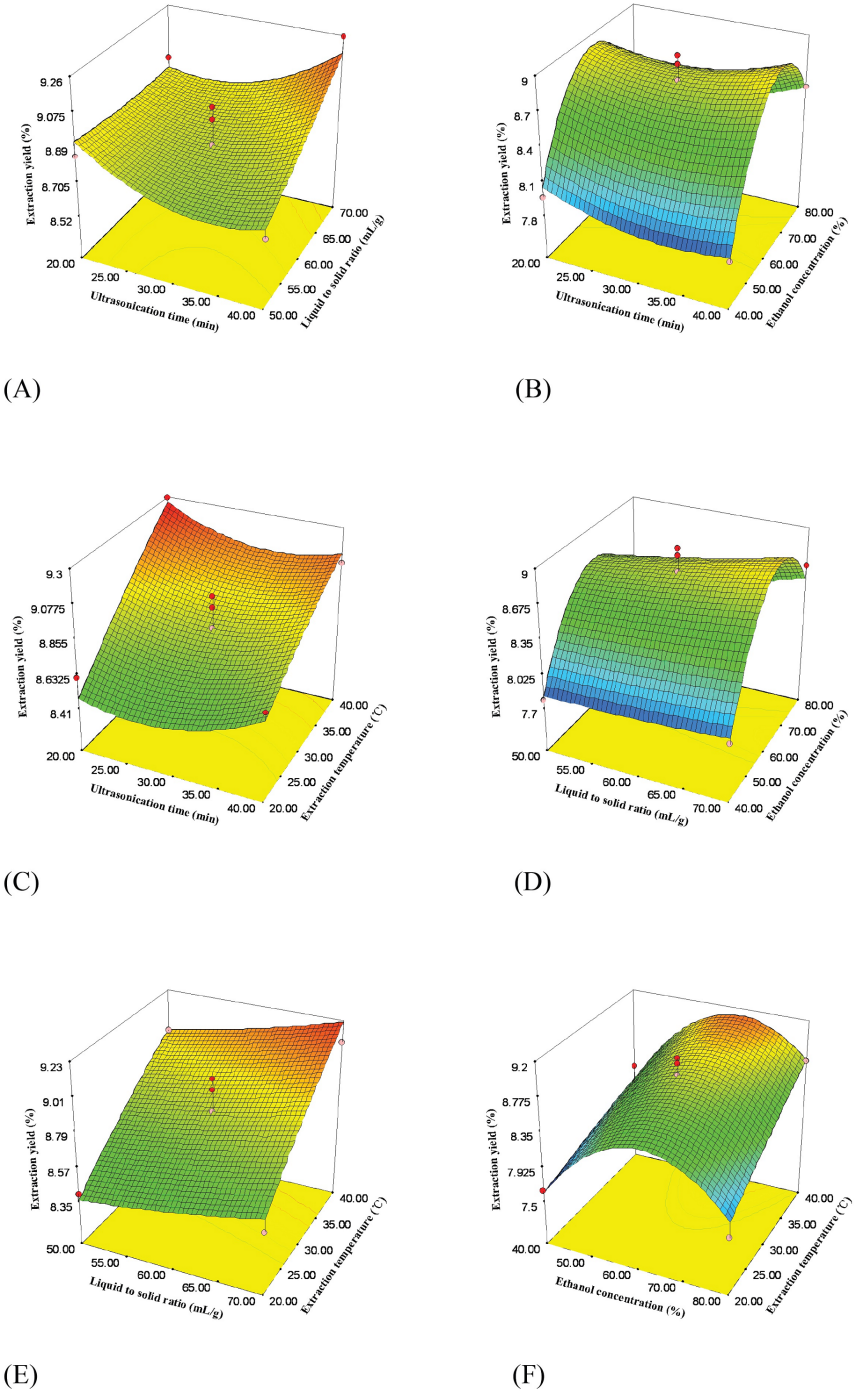
Response surface plots showing effects of extraction parameters on extraction yield of flavonoids. (A) Ultrasonication time vs. liquid to solid ratio (ethanol concentration = 60%, extraction temperature = 30 °C); (B) Ultrasonication time vs. ethanol concentration (liquid to solid ratio = 60 ml/g, extraction temperature = 30 °C); (C) Ultrasonication time vs. extraction temperature (ethanol concentration = 60%, liquid to solid ratio = 60 ml/g); (D) Liquid to solid ratio vs. ethanol concentration (ultrasonication time = 30 min, extraction temperature = 30 °C); (E) Liquid to solid ratio vs. extraction temperature (ultrasonication time = 30 min, ethanol concentration = 60%); (F) Ethanol concentration vs. extraction temperature (ultrasonication time = 30 min, liquid to solid ratio = 60 ml/g).

The relationship between every two variables is illustrated by 3D response surface plots (Fig. 2) and contour plots (Fig. 1A). As seen in Fig. 2A, when ultrasonication time was less than 30 min, liquid to solid ratio hardly affected extraction yield of *Epimedium* flavonoids. However, when ultrasonication time exceeded 30 min, extraction yield increased from 8.71% to 9.17% with an increase of liquid to solid ratio. Figure 2B show 3D response surface plot achieved by varying ultrasonication time and ethanol concentration and fixing liquid to solid ratio (0 level) and extraction temperature (0 level), respectively. Figure 2D illustrate 3D response surface plot with respect to liquid to solid ratio and ethanol concentration, respectively. Extraction yield at first rose and then declined as ethanol concentration increased (Figs. 2B and 2D). When ethanol solution was at the lower concentration (<55%), extraction yield was almost level with the increase of liquid to solid ratio. When ethanol solution was at the higher concentration (>55%), extraction yield increased markedly (Fig. 2D). The interaction between ultrasonication time and ethanol concentration exhibited the similar trend (Fig. 2B). With the increase of extraction temperature from 20 to 40 °C (liquid to solid ratio = 60 ml/g, ethanol concentration = 60% and ultrasonication time = 20 min), extraction yield rose from 8.47% to 9.27% (Fig. 2C). As shown in Fig. 2E, an increase of extraction temperature from 20 to 40 °C (ultrasonication time = 30 min, ethanol concentration = 60% and liquid to solid ratio = 70 ml/g) promoted the improvement of extraction yield from 8.55% to 9.22%. At extraction temperature below 30 °C, extraction yield slightly increased with the increase of liquid to solid ratio. However, at 40 °C, an increase in liquid to solid ratio from 50 to 70 ml/g caused a considerable increase of extraction yield from 8.97% to 9.22% (Fig. 2E). Figures 2B, 2D and 2F indicate the quadratic effect of ethanol concentration on extraction yield. It appears that ethanol concentration is a significant factor (Fig. 2F).

The software used for RSM generated the condition for recovery of flavonoids from *E. pubescens*: ultrasonication time of 39.71 min, liquid to solid ratio 69.41 ml/g, ethanol concentration 63.40%, and extraction temperature 37.98 °C. And the predicted extraction yield of flavonoids was 9.40%. Taking into account the operational feasibility and simplicity, the generated condition was slightly modified: ultrasonication time of 39 min, liquid to solid ratio 70 ml/g, ethanol concentration 63%, and extraction temperature 38 °C. In order to confirm the effectiveness of the modified condition, ultrasonic extraction was performed under the modified condition. The average extraction yield was found to be 9.44%, which was very close to the predicted value (9.40%) and was higher than all of response values in 29 experimental runs in BBD (Table 2). The results provided evidence that the modified condition was the optimal one.

#### Comparison between ultrasonic extraction and heating extraction

Ultrasonic extraction was compared with classical heating extraction in terms of extraction yield and chemical composition (see below). The condition for ultrasonic extraction (with ultrasound) and heating extraction (without ultrasound) was as follows: extraction duration of 39 min, liquid to solid ratio 70 ml/g, ethanol concentration 63%, and extraction temperature 38 °C. Noticeably, extraction yield obtained by ultrasonic extraction (9.44 ± 0.04%) is higher than that by heating extraction (8.74 ± 0.13%) (*p* < 0.01).

#### Impact of ultrasound on particle size distribution

**Figure 3:**
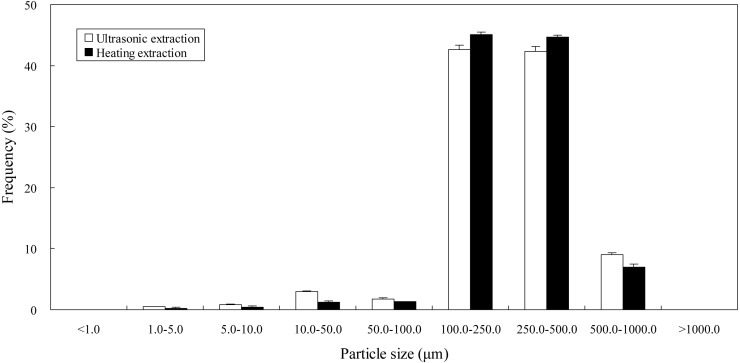
Particle size distribution of *Epimedium* samples processed by different extraction methods.

In order to identify the mechanism underlying ultrasonic extraction, particle sizes of *Epimedium* samples treated by ultrasonic extraction and heating extraction were determined by laser diffraction particle size analysis using deionized water as a dispersant. As shown in Fig. 3, frequency of small particle sizes (1.0–5.0, 5.0–10.0, 10.0–50.0 and 50.0–100.0 µm) and big particle sizes (500.0–1000.0 µm) of the samples processed by ultrasonic extraction was higher than that by heating extraction, while frequency of moderate particle sizes (100.0–250.0 and 250.0–500.0 µm) by ultrasonic extraction was lower than that by heating extraction. That is to say, with the assistance of ultrasound, the number of microparticles ranged from 1.0 to 100.0 µm (1.0–5.0, 5.0–10.0, 10.0–50.0 and 50.0–100.0 µm) in the samples most probably increases, whilst the number of macroparticles ranged from 100.0 to 500.0 µm (100.0–250.0 and 250.0–500.0 µm) most probably decreases. It is known that ultrasound may cause acoustic cavitation in the liquid. When the expanded bubbles or cavities collapsed, their potential energy would transform into kinetic energy in high-speed jets of liquid (approximately 400 km/h) (Luque-García & Luque de Castro, 2003). When *Epimedium* samples were stricken by high-speed jets of liquid, damage or ruptures might take place in the samples. The disruptions possibly resulted in the reduction of particle size of the samples and the improvement of their specific surface area. Thus the surface area for the sample-extractant contact increased drastically, which might aid in mass transfer between the samples and ethanol solution and hence increase extraction yield of flavonoids.

### Chemical composition of flavonoids from *E. pubescens*

**Figure 4:**
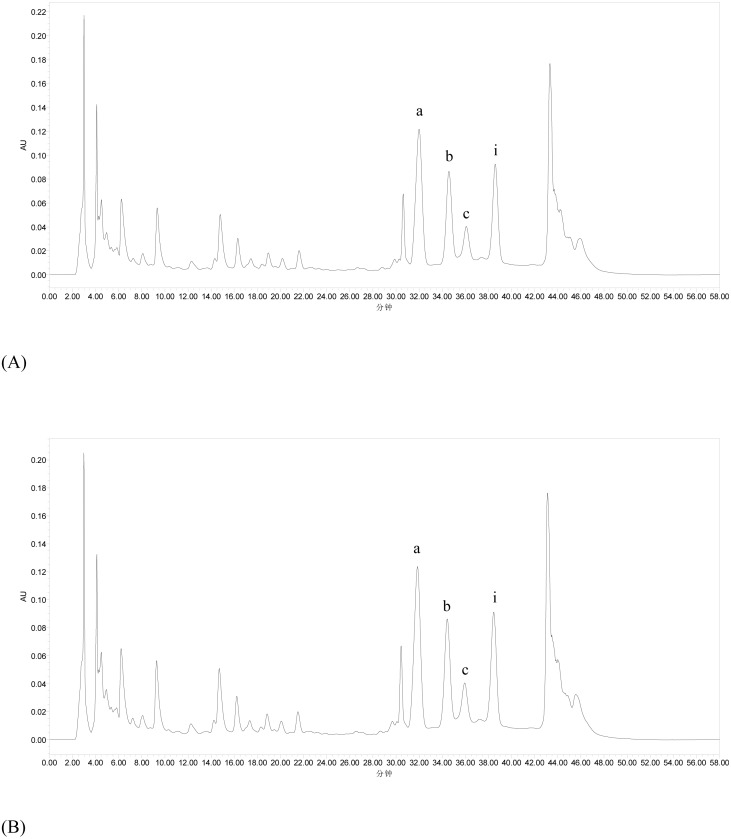
HPLC profiles of *Epimedium* flavonoids obtained by heating extraction (A) and ultrasonic extraction (B). Peaks a, b, c and i represent epimedin A, epimedin B, epimedin C and icariin, respectively. Peaks without a marker were not identified. The *x* and *y* coordinates represent retention time (min) and response value of HPLC-detector (AU), respectively.

Chemical characterization of flavonoids achieved by ultrasonic extraction and heating extraction is performed using HPLC method, and the typical chromatograms are shown in Fig. 4. HPLC analysis indicates that there are no significant peak differences between ultrasonic extraction and heating extraction, and peaks corresponding to icariin, epimedin A, epimedin B and epimedin C are observed in both extraction methods. Furthermore, contents of icariin and epimedin B in flavonoids obtained by ultrasonic extraction were remarkably higher than those by heating extraction (*p* < 0.05).

### DPPH^⋅^ and ABTS^⋅+^ radical-scavenging activities

**Figure 5:**
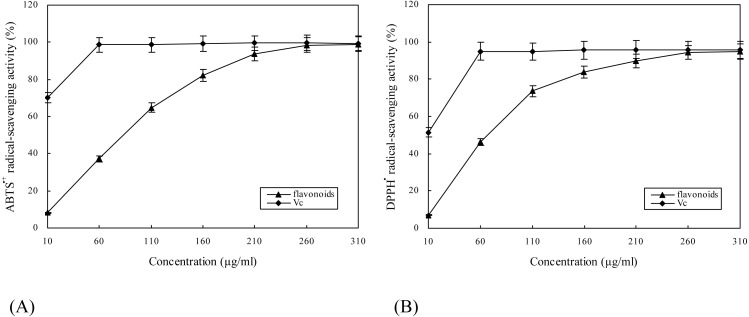
ABTS^⋅+^ (A)and DPPH^⋅^ (B) radical-scavenging activities of flavonoids from *E. pubescens*.

Two chemical-based approaches, DPPH and ABTS assays, were employed to evaluate *in vitro* antioxidant capacities of flavonoids isolated from *E. pubescens* by ultrasonic extraction. As seen in Fig. 5, flavonoids scavenged ABTS^⋅+^ and DPPH^⋅^ radicals in a dose-dependent manner. As regards ABTS^⋅+^ radicals, EC_50_ values for flavonoids and Vc were 55.8 and 0.5 µg/ml, respectively. As regards DPPH^⋅^ radicals, EC_50_ values for flavonoids and Vc were 52.1 and 4.7 µg/ml, respectively. At concentration of 260 µg/ml, ABTS^⋅+^ and DPPH^⋅^ radical-scavenging ability of flavonoids was sufficiently close to that of Vc (Fig. 5). These results imply that flavonoids obtained by ultrasonic extraction are an efficient scavenger of free radicals.

### Effects of flavonoids on activities of antioxidant enzymes in *D. melanogaster*

In order to further ascertain antioxidant capacities of flavonoids extracted from *E. pubescens*, effects of flavonoids on activities of CAT and GSH-Px in *D. melanogaster* were investigated on the basis of the above antiradical assay. The results were summarized in Table 3. Statistically, there were marked differences of GSH-Px activities between the experimental group and the control group (*p* < 0.05). CAT activities of female fruit flies fed the flavonoid medium were notably higher than those of female fruit flies fed the cornmeal medium (*p* < 0.05), and CAT activities of male fruit flies in the experimental group were slightly higher than those of male fruit flies in the control group (*p* = 0.05). For females, oral administration of flavonoids improved CAT and GSH-Px activities by 13.58% and 5.18%, respectively. For males, oral administration of flavonoids increased CAT and GSH-Px activities by 13.90% and 5.65%, respectively. Interestingly, gender differences in activities of antioxidant enzymes were observed when fruit flies were fed the flavonoid medium (Table 3). Similarly, there were sex differences in lifespan while fruit flies were fed the medium containing commercially available antioxidant supplements (Vrailas-Mortimer et al., 2012). The reason for these differences remains obscure. Overall, CAT and GSH-Px activities in fruit flies fed the flavonoid medium were higher than those in fruit flies fed the cornmeal medium (Table 3), implying that *Epimedium* flavonoids considerably affected antioxidant defense system in *D. melanogaster*. The findings are consistent with the results of ABTS and DPPH assays. That is to say, *Epimedium* flavonoids exhibit relatively high *in vitro* and *in vivo* antioxidant ability. Due to the fact that lots of synthetic antioxidants applied to food and drug industries have adverse health effects such as carcinogenicity, it is essential to seek new natural antioxidants (Shebis et al., 2013; Neha et al., 2019). This study suggests a potential role for *Epimedium* flavonoids as a new natural antioxidant capable of reducing the production of ROS.

**Table 3:** Effects of *Epimedium* flavonoids on CAT and GSH-Px activities in *D.melanogaster*.

Gender	CAT activity (U/mg protein)^a^		Significance^b^	GSH-Px activity (U/mg protein)^a^		Significance^b^
	*Epimedium* flavonoids	Control		*Epimedium* flavonoids	Control	
Female	34.21 ± 0.62	30.12 ± 0.81	s	178.54 ± 1.57	169.74 ± 1.39	s
Male	49.06 ± 1.35	43.07 ± 1.41		204.71 ± 2.63	193.77 ± 2.14	s
All^c^	41.64 ± 0.98	36.60 ± 1.11	s	191.62 ± 2.10	181.76 ± 1.77	s

**Notes.**

a mean ± SD

b s represents significant (0.01 ≤ *p* < 0.05).

c female + male.

## Conclusions

The condition for extraction of antioxidant flavonoids from *E. pubescens* was optimized using response surface methodology. Ultrasonic extraction most probably decreased particle sizes of *Epimedium* samples, thus facilitating mass transfer between the samples and ethanol solution. Four flavonoids, icariin, epimedin A, epimedin B and epimedin C, were identified from *Epimedium* flavonoids. *Epimedium* flavonoids possessed ABTS^⋅+^ and DPPH^⋅^ radical-scavenging capacities. Consistent with their antiradical ability, oral administration of *Epimedium* flavonoids markedly improved CAT and GSH-Px activities in the model organism *D. melanogaster*. Flavonoids ultrasonically extracted from *E. pubescens* shows great potential for becoming a natural antioxidant owing to their antiradical ability and effects on CAT and GSH-Px of *D. melanogaster*.

##  Supplemental Information

## Additional Information and Declarations

### Data Availability

The following information was supplied regarding data availability: The raw measurements are available in the Supplemental Files.
